# Supplementary material for: Crawling and Gliding: A Computational Model for Shape-Driven Cell Migration
Source: PLoS Comput Biol. 2015 Oct 21;11(10):e1004280. doi: 10.1371/journal.pcbi.1004280 (PMC4619082; doi:10.1371/journal.pcbi.1004280)
Supplement: S1 Code — (ZIP) [file pcbi.1004280.s012.zip › release/tst/doc/html/dish_8h_source.html]

Tissue Simulation Toolkit: dish.h Source File


Tissue Simulation Toolkit
 0.1.4.1


- Main Page
- Namespaces
- Classes
- Files

- File List
- File Members

dish.h

Go to the documentation of this file.

1 /\*

2

3 Copyright 1996-2006 Roeland Merks

4

5 This file is part of Tissue Simulation Toolkit.

6

7 Tissue Simulation Toolkit is free software; you can redistribute

8 it and/or modify it under the terms of the GNU General Public

9 License as published by the Free Software Foundation; either

10 version 2 of the License, or (at your option) any later version.

11

12 Tissue Simulation Toolkit is distributed in the hope that it will

13 be useful, but WITHOUT ANY WARRANTY; without even the implied

14 warranty of MERCHANTABILITY or FITNESS FOR A PARTICULAR PURPOSE.

15 See the GNU General Public License for more details.

16

17 You should have received a copy of the GNU General Public License

18 along with Tissue Simulation Toolkit; if not, write to the Free

19 Software Foundation, Inc., 51 Franklin St, Fifth Floor, Boston, MA

20 02110-1301 USA

21

22 \*/

23

30 #ifndef CRITTER\_H\_

31 #define CRITTER\_H\_

32 #include <vector>

33 #include "graph.h"

34 #include "random.h"

35 #include "pde.h"

36 #include "cell.h"

37 #include "ca.h"

38

39 namespace ColourMode {

40  enum { State,CellType,Sigma,Auxilliary };

41 }

42

43 class Dish {

44

45  friend class Info;

46

47 public:

48  Dish(void);

49

57  void Init(void);

58

59  void ConstructorBody(void);

60

61  virtual ~Dish();

62

67  void Plot(Graphics \*g);

68

69

70  int ZygoteArea(void) const;

71

73  int Time(void) const;

74

76  int CountCells(void) const;

77

84  void CellGrowthAndDivision(void);

85

87  int Area(void) const;

88

90  int TargetArea(void) const;

91

93  int SizeX(void);

94

96  int SizeY(void);

97

99  inline Cell &getCell(int c) {

100  return cell[c];

101  }

102

103  PDE \*PDEfield;

104  CellularPotts \*CPM;

105

106  // Was used for gradient measurements, not functional now.

107  void ClearGrads(void);

108

109  void MeasureChemConcentrations(void);

110 protected:

112  void SetCellOwner(Cell &which\_cell);

113

114 private:

115  bool CellLonelyP(const Cell &c, int \*\*neighbours) const;

116

117 protected:

119  std::vector<Cell> cell;

120 };

121

122 #define INIT void Dish::Init(void)

123

124 #endif

pde.h

Cell

**Definition:** cell.h:33

ColourMode::State

**Definition:** dish.h:40

Dish::MeasureChemConcentrations

void MeasureChemConcentrations(void)

**Definition:** dish.cpp:223

ca.h

Dish::~Dish

virtual ~Dish()

**Definition:** dish.cpp:64

Dish::ConstructorBody

void ConstructorBody(void)

**Definition:** dish.cpp:77

Dish::Init

void Init(void)

Init defines the initial state of the virtual cell culture.

ColourMode::Auxilliary

**Definition:** dish.h:40

Dish::CellGrowthAndDivision

void CellGrowthAndDivision(void)

Stretched induced cell growth and division.

**Definition:** dish.cpp:113

Dish::Dish

Dish(void)

**Definition:** dish.cpp:43

CellularPotts

**Definition:** ca.h:57

Dish::ClearGrads

void ClearGrads(void)

**Definition:** dish.cpp:205

cell.h

PDE

**Definition:** pde.h:31

Dish::cell

std::vector< Cell > cell

The cells in the Petri dish; accessible to derived classes.

**Definition:** dish.h:119

Dish::TargetArea

int TargetArea(void) const

Returns the summed of all cells target area in the dish.

**Definition:** dish.cpp:181

Dish

The virtual Petri dish.

**Definition:** dish.h:43

ColourMode::Sigma

**Definition:** dish.h:40

Dish::Plot

void Plot(Graphics \*g)

Plot the Dish to graphics window g.

**Definition:** dish.cpp:71

Dish::getCell

Cell & getCell(int c)

Returns a reference to cell number "c".

**Definition:** dish.h:99

graph.h

Dish::CountCells

int CountCells(void) const

Returns the number of cells in the dish, excluding apoptosed cells.

**Definition:** dish.cpp:150

Graphics

API for Graphics windows.

**Definition:** graph.h:36

Dish::SizeX

int SizeX(void)

Returns the horizontal size of the dish.

**Definition:** dish.cpp:252

random.h

Dish::CPM

CellularPotts \* CPM

**Definition:** dish.h:104

ColourMode::CellType

**Definition:** dish.h:40

Dish::ZygoteArea

int ZygoteArea(void) const

**Definition:** dish.cpp:214

Dish::Area

int Area(void) const

. Returns the summed area of all cells in the dish

**Definition:** dish.cpp:166

Dish::PDEfield

PDE \* PDEfield

**Definition:** dish.h:103

Dish::SizeY

int SizeY(void)

Returns the horizontal size of the dish.

**Definition:** dish.cpp:253

Info

Enables interactive querying of the simulation.

**Definition:** info.h:36

Dish::SetCellOwner

void SetCellOwner(Cell &which\_cell)

Assign a the cell to the current Dish.

**Definition:** dish.cpp:199

Dish::Time

int Time(void) const

Returns the number of completed Monte Carlo Steps.

**Definition:** dish.cpp:218


---

Generated on Thu Aug 14 2014 22:04:01 for Tissue Simulation Toolkit by  

 1.8.6
